# Translocation of Foliar Absorbed Zn in Sunflower (*Helianthus annuus*) Leaves

**DOI:** 10.3389/fpls.2022.757048

**Published:** 2022-03-02

**Authors:** Cui Li, Linlin Wang, Jingtao Wu, F. Pax C. Blamey, Nina Wang, Yanlong Chen, Yin Ye, Lei Wang, David J. Paterson, Thea L. Read, Peng Wang, Enzo Lombi, Yuheng Wang, Peter M. Kopittke

**Affiliations:** ^1^School of Ecology and Environment, Northwestern Polytechnical University, Xi’an, China; ^2^Key Laboratory of Vegetation Restoration and Management of Degraded Ecosystems, South China Botanical Garden, Chinese Academy of Sciences, Guangzhou, China; ^3^School of Agriculture and Food Sciences, The University of Queensland, Brisbane, QLD, Australia; ^4^Australian Synchrotron, Clayton, VIC, Australia; ^5^Future Industries Institute, University of South Australia, Mawson Lakes, SA, Australia; ^6^College of Resources and Environmental Sciences, Nanjing Agricultural University, Nanjing, China

**Keywords:** foliar fertilizers, translocation, sunflower, XFM, Zn nutrition

## Abstract

Foliar zinc (Zn) fertilization is an important approach for overcoming crop Zn deficiency, yet little is known regarding the subsequent translocation of this foliar-applied Zn. Using synchrotron-based X-ray fluorescence microscopy (XFM) and transcriptome analysis, the present study examined the translocation of foliar absorbed Zn in sunflower (*Helianthus annuus*) leaves. Although bulk analyses showed that there had been minimal translocation of the absorbed Zn out of the leaf within 7 days, *in situ* analyses showed that the distribution of Zn in the leaf had changed with time. Specifically, when Zn was applied to the leaf for 0.5 h and then removed, Zn primarily accumulated within the upper and lower epidermal layers (when examined after 3 h), but when examined after 24 h, the Zn had moved to the vascular tissues. Transcriptome analyses identified a range of genes involved in stress response, cell wall reinforcement, and binding that were initially upregulated following foliar Zn application, whereas they were downregulated after 24 h. These observations suggest that foliar Zn application caused rapid stress to the leaf, with the initial Zn accumulation in the epidermis as a detoxification strategy, but once this stress decreased, Zn was then moved to the vascular tissues. Overall, this study has shown that despite foliar Zn application causing rapid stress to the leaf and that most of the Zn stayed within the leaf over 7 days, the distribution of Zn in the leaf had changed, with Zn mostly located in the vascular tissues 24 h after the Zn had been applied. Not only do the data presented herein provide new insight for improving the efficiency of foliar Zn fertilizers, but our approach of combining XFM with a transcriptome methodological system provides a novel approach for the study of element translocation in plants.

## Introduction

Zinc (Zn) is a micronutrient for both plants and humans. However, Zn deficiency ranks as the third most common micronutrient deficiency in humans (Menguer et al., 2018), with more than 30% of the global human population suffering from Zn deficiency (Xia et al., 2020). This deficiency in the global human population is primarily due to the low dietary Zn supply caused by widespread crop Zn deficiency (Zhang et al., 2018). Foliar fertilization with Zn is an efficient approach to overcome crop Zn deficiency and to fortify foods with Zn, thus alleviating human Zn deficiency, especially for soils that limit the root uptake of Zn (Joy et al., 2015; Fu et al., 2016). However, much remains unknown regarding the translocation of the Zn in crops following its initial movement across the leaf surface.

We have previously examined the absorption of foliar-applied Zn in sunflower (*Helianthus annuus*), finding that Zn is absorbed through the base of non-glandular trichomes (NGTs) and the general cuticular area (i.e., leaf areas covered by the cuticle without stomata and trichomes) (Li et al., 2019). However, following its absorption, the subsequent movement of Zn (i.e., translocation) is considered to be limited in sunflower (Li et al., 2017) and also in other plant species (Zhang and Brown, 1999; Du et al., 2015). How Zn moves within the leaf following its absorption, and the factors regulating this movement, remain unclear. This lack of knowledge hinders the optimal use of Zn foliar fertilizers and the development of new and more efficient Zn foliar fertilizers.

Theoretically, the translocation of foliar absorbed Zn involves three processes: (1) movement of the Zn from the apoplast of the leaf epidermal cells to the leaf vascular tissues (phloem and xylem); (2) long-distance translocation within the vascular tissues; and (3) unloading from the vascular tissues and translocation into the destination tissues. It has been proposed that the limited translocation of foliar-absorbed Zn could be due to several factors, including the strong binding of Zn within the leaf epidermal cell wall (Du et al., 2015), low mobility within the phloem (Wu et al., 2010; Xue et al., 2015), the use of different Zn fertilizer types that differ in their properties (Doolette et al., 2018), or differences in the phenological stages of the plant that alter the translocation of the Zn (Fernández and Brown, 2013). Furthermore, it has been reported that Zn deficiency promotes the redistribution of Zn from old leaves to young leaves (Xie et al., 2019). However, few studies have utilized molecular biology to investigate the foliar translocation of Zn in order to understand the mechanisms that regulate its translocation.

In the present study, we use two broad approaches for improving our understanding of the translocation of foliar absorbed Zn. Firstly, it is useful to examine progressive changes in the distribution of elements in plants. In this regard, synchrotron-based X-ray fluorescence microscopy (XFM) is a valuable tool that can be used for obtaining maps showing the real-time element distribution in living plants (Blamey et al., 2018; Kopittke et al., 2020). Secondly, RNA-sequence analysis is a powerful technique for understanding gene expression. The present study combined these two approaches, allowing us to relate the changes in Zn distribution in the leaf to the underlying gene expression. Using sunflower, we first examined the *in situ* distribution of Zn in the leaf in a time-resolved manner using synchrotron-based XFM in living plants. Next, based upon the distribution of Zn, RNA-sequence analysis was employed to show the leaf gene expression at different Zn translocation stages. This study provides novel information on the movement of foliar absorbed Zn within the leaf and corresponding changes in leaf gene expression during this process. It is expected that the results will increase our understanding of the translocation of foliar Zn fertilizers and provide new insight in improving the efficiency of Zn foliar fertilizers, thus assisting to alleviate the health problems caused by Zn deficiency in 30% of the global human population.

## Materials and Methods

### General Conditions for Plant Growth

Sunflower (cv. Hyoleic 41) seeds were germinated in rolled paper towels covered with a plastic bag placed vertically in a beaker filled with tap water for 4 days. Thereafter, four seedlings were transferred to black buckets (11 L) using the same method and nutrient solution composition as described in Li et al. (2018a). Briefly, the plants were grown at a temperature of 25°C and with artificial light (photon flux density of 1,500 μmol m^–2^s^–1^) for 12 h d^–1^. Nutrient solutions were continuously aerated and changed weekly. Aliquots (5 ml) of 44 mM KH_2_PO_4_ were supplied to each bucket every other day after 10 days of growth to replace the phosphorus (P) being removed by plant growth.

### Fertilizer Application and Bulk Leaf Zn Concentrations

The Zn foliar fertilizer used in the study was 1,000 mg Zn L^–1^ (15.4 mM, pH 5.2) with 0.05% Tween 20 (Reuveni et al., 1994), with the solution prepared using ZnSO_4_. 7H_2_O. Using 2-week-old sunflower plants, Zn was applied using a pipette as 5 μL droplets onto the youngest fully expanded leaf (YFEL) surface, with each leaf having a total of 30 droplets. The Zn was always applied to the adaxial leaf surface. After 0.5 h, all the droplets were removed by blotting dry using filter paper, with the leaf still attached to the plants. Then, the leaf blades were cut from the petioles at 0, 6, 24, 60, and 168 h after removing the droplets. Leaves to which no Zn was applied were also harvested at 0 and 168 h as controls. All the leaves were then rinsed thoroughly using (sequentially) deionized water, 3% ethanol, 2% HNO_3_, and then deionized water (Vu et al., 2013). Then the leaves were dried at 60°C and digested using a 5:1 mixture of nitric acid and perchloric acid. We measured Zn concentrations using atomic absorption spectrometry, with blanks and reference standards included to ensure accuracy. Three replicates were utilized.

### Tracing Zn Distribution in Sunflower Leaf Using X-ray Fluorescence Microscopy

This experiment was conducted at the XFM beamline of the Australian Synchrotron (Clayton, Australia) (Paterson et al., 2011; Howard et al., 2020). The excitation energy was 12,900 eV, the X-rays were selected using a Si (111) monochromator, and the beam size was 2 × 2 μm. The total photon flux was ca. 2.9 × 10^9^ photons s^–1^ on the sample. A 384-element Maia detector system in a backscatter position of the sample was used to collect the X-ray fluorescence emitted by the specimen (Kopittke et al., 2011). Two types of analyses were conducted using XFM: (1) Repeated, time-resolved scans using living plants to examine changes in Zn distribution in leaves following the foliar application of Zn (Blamey et al., 2018); and (2) scans using freeze-dried cross-sections harvested at various times after applying the Zn (Li et al., 2018a).

For the first analysis (time-resolved scans in living plants), 2-week-old sunflower plants were transferred to the Australian Synchrotron and individual plants were placed in a 50 ml centrifuge tube with the stem held firmly in place using foam. The tube (and plant) was placed in a customized sample holder, with the YFEL mounted flat onto a sample window with the adaxial surface of the leaf (i.e., the surface to which the Zn had been applied) facing the incident beam and the detector. The tube was filled with nutrient solution and the plant was maintained in artificial light (as described above) unless being scanned. A 5 μL droplet of the Zn fertilizer solution was applied to the YFEL using a pipette. After 0.5 h, the droplet was removed by blotting dry using filter paper. From the time when the Zn was removed from the leaf surface, the leaf was scanned repeatedly at 0.25, 2.2, 4, 6, 20, 45, and 60 h. Two replicate leaves were examined (the leaves being on different plants), with the second leaf scanned after 1, 3, 5, 7, 21, 46, and 61 h. For each scan, a coarse scan was first conducted to locate the area where the Zn had initially been applied and to ensure that each scan was performed on the same area. After this initial coarse scan, a fine scan was conducted to obtain a higher resolution map. The coarse scan was conducted at a step size of 30 μm and a horizontal stage velocity of 5 mm s^–1^, resulting in a pixel transit time of 6 ms. The fine scan was done at a step size of 2 μm and horizontal stage velocity of 1.5 mm s^–1^, resulting in a pixel transit time of 1.33 ms. The XFM data were analyzed using GeoPIXE (Ryan and Jamieson, 1993; Ryan, 2000). Following completion of the experimental period, the leaves were visually examined and showed no damage.

For the second type of analysis, the freeze-dried leaf sections were prepared in advance. To provide a larger area that could be sectioned, a 100 μL Zn droplet was applied to the YFELs of 40-day-old sunflower plants for 0.5 h and then removed by blotting dry using filter paper. Once the droplet had been removed, the leaves were then excised after 0, 0.5, 3, and 24 h. YFELs that had not received any Zn were used as the control. Following excision, the YFELs were rinsed thoroughly using deionized water, 3% ethanol, 2% HNO_3_, and deionized water (Vu et al., 2013). The area where Zn had been applied was excised, embedded in 4% agar and sections (150 μm in thickness) prepared using a vibratome (Leica VT 1000s) (Li et al., 2018b). The sections were then freeze-dried after sealing between Ultralene films (holes were punched in the film to aid the freeze-drying process) which were held using XRF sample cups (SC-8047, Premier Lab Supply). The freeze-dried samples were then transferred to the Australian Synchrotron with the sample cup for analysis. At the Australian Synchrotron, the sections were placed between Ultralene films (4 μm thickness) on the sample holder (Supplementary Figure 1). Two replicates were examined. A rapid coarse scan was first used to locate the samples before a fine scan was performed at a higher resolution. The parameters of the scans were the same as indicated earlier.

### Transcriptome Analysis of Leaf During Different Zn Translocation Stages

Based on the XFM analyses (see section “Results”), the Zn distribution in the leaf cross-sections differed markedly for the samples examined 0 and 24 h after the initial 0.5 h application period, with the overall Zn translocation rate increasing at ca. 20 h. Therefore, the following samples were collected for transcriptome analysis: (1) The YFELs without Zn treatment as control (L0); (2) the YFELs to which Zn applied for 0.5 h before being removed and then harvested immediately as L1 (i.e., 0 h after removing the Zn); and (3) the YFELs to which Zn applied for 0.5 h and grown for 24 h after fertilizer removal as L2 (i.e., 24 h after removing the Zn). For this experiment, Zn was sprayed over the entire leaf surface. The leaves were excised at the required harvest time and rinsed using deionized water. The samples were placed into RNase-free tubes and immediately frozen in liquid nitrogen and placed in a freezer (–80°C) until further analysis. Three biological replicates were examined.

Total RNA was extracted using the RNAprep pure plant kit (DP441, Tiangen) following the manufacturer’s instructions. The RNA quantification and quality were examined using 1% agarose gels, a NanoPhotometer spectrophotometer (IMPLEN, CA, United States), and the RNA Nano 6000 Assay Kit of the Bioanalyzer 2100 system (Agilent Technologies, CA, United States). Next, 1 μg RNA per sample was used to generate the RNA sequencing libraries using NEBNext Ultra RNA Library Prep Kit for Illumina (Novogene, Beijing, China) following the manufacturer’s instructions. The clustering of the samples was performed using a cBot Cluster Generation System with TruSeq PE Cluster Kit v3-cBot-HS (Illumina). The sequencing analysis was then conducted on an Illumina NovaSeq platform after cluster generation and 150 bp paired-end reads were generated. After obtaining the raw reads, the reads of low quality and containing adapter or poly-N were removed to get the clean reads, with the Q20, Q30, and guanine and cytosine (GC) content of the clean reads calculated. All analyses were performed using the processed clean reads. The clean reads were aligned to the genome of the sunflower using Hisat2 v2.0.5 (Badouin et al., 2017).

For the gene expression quantification, the read numbers of each gene were counted using featureCounts v1.5.0-p3, and the fragments per kilobase of transcript per million mapped reads (FPKM) of each gene were calculated for comparison among different samples (Nautiyal et al., 2020). The DESeq2 R package was employed for analyzing the differentially expressed genes (DEGs) between the three samples, i.e., genes with an adjusted *p*-value (*p*adj) ≤ 0.05 and | log2(FoldChange) | ≥ 0 were assigned as differentially expressed. Gene Ontology (GO) and Kyoto Encyclopedia of Genes and Genomes (KEGG) analyses were used to classify the functions of the DEGs by using the clusterProfiler R package (3.4.4), with *p*adj < 0.05 identified as significantly enriched GO terms and *p*-value < 0.05 as KEGG pathways.

### Quantitative Reverse Transcription PCR Validation

To validate the transcriptome results, 15 genes were selected for Quantitative Reverse Transcription PCR (RT-qPCR) analysis. The total RNA was extracted as described above. The first-strand complementary DNA (cDNA) was synthesized using the Prime Script^®^ RT reagent Kit (Takara, Japan). The primers were designed using Primer3 (v. 0.4.0) (Supplementary Table 1). RT-qPCR was performed using an SYBR Premix EX Taq Kit (Takara) in a 20 μL reaction mixture on an ABI7300 (Applied Biosystems, United States). The Actin (gene ID: 110903735) and Ubiquitin (gene ID: 110936586) were used as the internal controls (Fernandez et al., 2008; Ramu et al., 2016). The relative expression was assessed using the 2^–ΔΔCT^ method (Livak and Schmittgen, 2001).

## Results

### Changes in Leaf Bulk Zn Concentration After Foliar Zn Application

**FIGURE 1:**
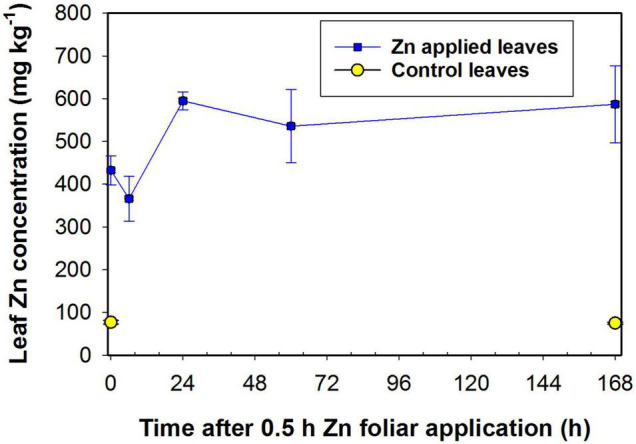
Bulk concentration of zinc (Zn) in sunflower leaves to which Zn had been foliar-applied. The Zn was applied for 0.5 h before then being removed, leaves were then harvested after growing for a further 0, 6, 24, 60, and 168 h. For the control leaves, no Zn was applied and the leaves were harvested after 0 and 168 h. Data are mean ± SD (*n* = 3).

Following the initial application of Zn for 0.5 h, the concentration of Zn within the bulk leaf tissue (ca. 400–600 mg kg^–1^) was substantially higher than for the control (ca. 100 mg kg^–1^) (Figure 1). For the leaves to which Zn had been applied, the slight increase in the leaf Zn concentration from 0 to 24 h was possibly due to the continued absorption of Zn from the residual Zn on the leaf surface given that the Zn was only removed using a filter paper after 0.5 h. It is noteworthy that there was no notable decrease in the bulk leaf tissue Zn concentration over time, suggesting that there was not a pronounced translocation of Zn out of the Zn applied leaf.

### *In situ* Analyses of Foliar Absorbed Zn in Sunflower Leaves

**FIGURE 2:**
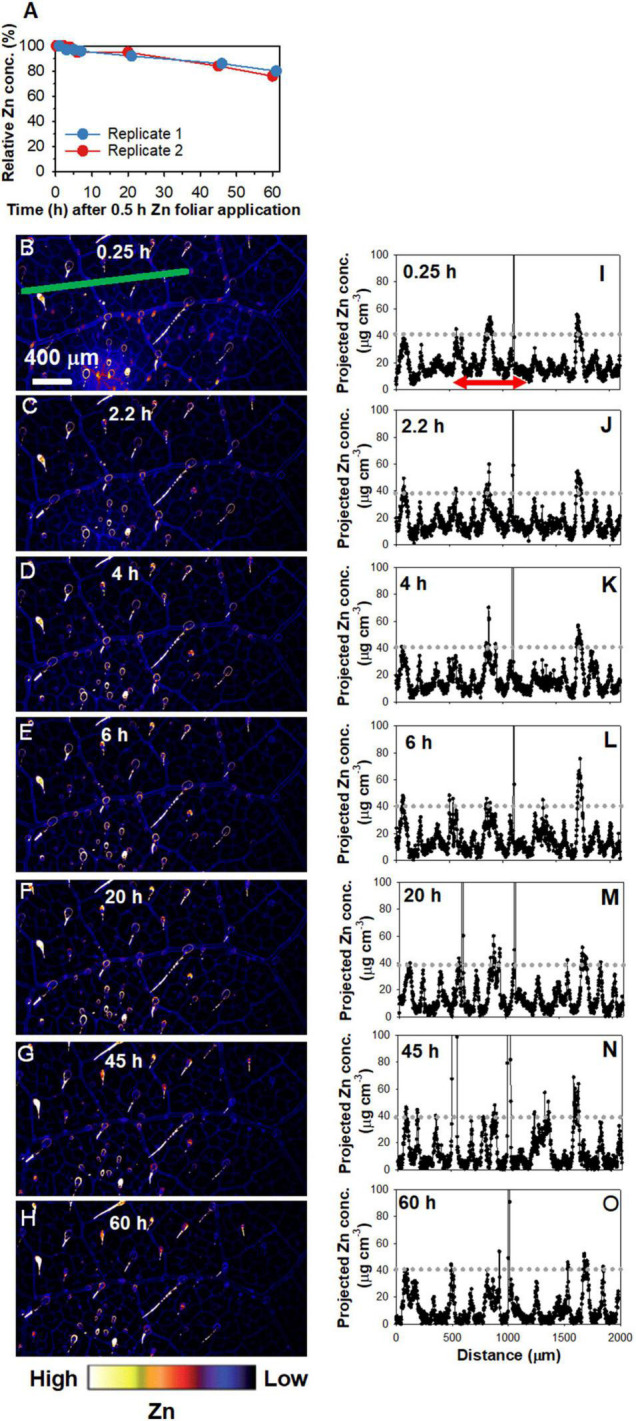
Repeated X-ray fluorescence microscopy (XFM) scans of the same leaf area of sunflower over 60 h showing the changes in the Zn concentration under the area previously exposed to a 5 μL droplet of 1,000 mg Zn L^– 1^ (15.4 mM, pH 5.2) with 0.05% Tween for 0.5 h. Note that the entire area in the images shown was from the area beneath the area to which the Zn had been applied. **(A)** Changes in the relative Zn concentrations with time, calculated using the average Zn concentrations of the areas in **(B–H)** and Supplementary Figure 2. (i.e., two replicates). **(B–H)** Maps showing the Zn distribution for Replicate 2, with the scans conducted at 0.25 h **(B)**, 2.2 h **(C)**, 4 h **(D)**, 6 h **(E)**, 20 h **(F)**, 45 h **(G)**, and 60 h **(H)** following 0.5 h foliar application. Part of **(B–H)** is from Li et al. (2021) with using permission. **(I–O)** showing the projected Zn concentration along the green line in **(B–H)**, respectively. The red arrow in **(I)**, which applies to **(J–O)** also, allows a comparison of Zn concentration in the veins and interveinal area. Colors are comparable in **(B–H)**. The scale in B applies to **(C–H**).

To allow for *in situ* analyses of the changes in the distribution of Zn following its foliar absorption, time-resolved XFM analyses using living plants were used to examine the leaf areas where the Zn had been applied for 0.5 h and then removed (Figure 2 and Supplementary Figure 2). Importantly, when examining these elemental distribution maps, it must be noted that the Zn was applied as a single droplet and that the entire area is shown in Figure 2 and Supplementary Figure 2 is from an area originally under droplet. Firstly, changes in the relative average Zn concentrations of the area were analyzed (Figure 2A). It was found that the two replicates had a similar trend, with the relative Zn concentration decreasing by ca. 5% after 20 h, and then decreasing by ca. 20% after 60 h. Indeed, when comparing the same leaf scanned repeatedly over time, the decreased brightness of colors from 0.25 to 60 h showed that the concentration of Zn in the leaf tissues underlying the area where the Zn had been applied gradually decreased over time (Figures 2B–H and Supplementary Figure 2). Particularly, the roles of NGTs in absorption and translocation of foliar-applied Zn are not discussed here as it has been discussed previously in Li et al. (2019, 2021).

Next, the transect indicated by the green line in Figures 2B–H was used to examine the Zn concentration at a finer scale over time (Figures 2I–O). The initial peaks at ca. 40 μg cm^–3^ in Figures 2I–O showed that the highest concentration of Zn along this linear transect corresponded to the veins. In contrast, the interveinal areas had ca. 10 μg cm^–3^ Zn—a concentration that decreased as time progressed to typically < 5 μg cm^–3^ after 20 h, and continued decrease thereafter (Figures 2I–O). This suggests that the Zn in the interveinal areas was gradually translocated away, most likely to the veins.

**FIGURE 3:**
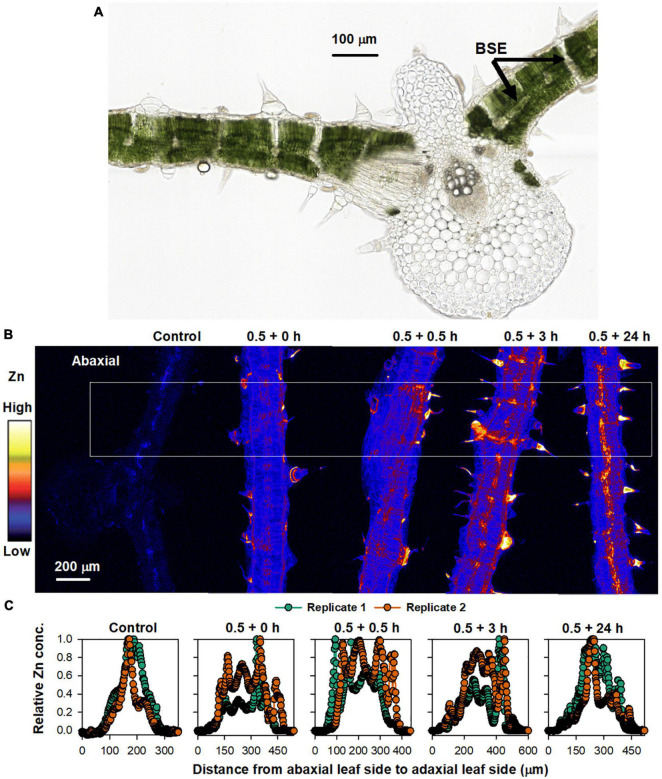
Sunflower leaves to which Zn had been applied. **(A)** Light micrograph of sunflower leaf cross-section, showing the bundle sheath extension (BSE) (from Li et al., 2018b with permission). **(B)** XFM images showing Zn distribution in cross-sections of a control leaf, and leaves to which Zn had been applied for 0.5 h before the Zn was removed and the plant then grown for a further 0 (0.5 + 0 h), 0.5 (0.5 + 0.5 h), 3 (0.5 + 3 h), and 24 h (0.5 + 24 h) (all leaf tissues shown were sampled from underneath where the Zn had applied). The colors are comparable in **(B)**. **(C)** Relative average Zn concentration along each leaf area from the white box in **(B)**. Two replicates were examined, with Replicate 1 shown here and Replicate 2 shown in Supplementary Figure 3.

To further assess the changes in Zn concentrations in the leaf over time, we examined the Zn distribution in leaf cross-sections that were collected 0, 0.5, 3, and 24 h following the initial 0.5 h Zn application (Figure 3). Light microscopy showed that bundle sheath extensions (BSEs) connected the leaf tissues both vertically and horizontally, with the vertical BSEs generally connecting with NGTs on adaxial and abaxial surfaces and the horizontal BSEs connecting to the leaf veins (Figure 3A). In this regard, it is known that the BSEs play an important role in the translocation of foliar absorbed Zn (Li et al., 2019). As expected, XFM analysis showed that Zn concentration within the leaf tissues was higher for all treated leaves than for the control in both replicates. However, the distribution of the Zn within the leaf cross-sections changed over time (Figures 3B,C and Supplementary Figure 3). We examined average Zn concentrations along a linear transect that was ca. 500 μm wide across the five treatments as shown by the white box in Figure 3B, with these average values shown in Figure 3C. For the control leaf, the highest Zn concentration was in the middle (inner) tissues of the leaf. Immediately after Zn application (i.e.,0.5 + 0 h), the highest Zn concentration was found in the adaxial epidermal layer (Zn having been applied to the adaxial leaf surface). It was interesting that in as little as 0.5 h since the initial application of foliar Zn, some of the Zn that had been absorbed was already located in the inner leaf tissues and in the abaxial epidermal layer. This raises the possibility of Zn being translocated from the adaxial surface to the abaxial surface by the vertical BSEs (Li et al., 2019). After 3 h (0.5 + 3 h), there was a pronounced movement of Zn across the entire cross-section, including in the BSEs and the NGT bases. After 24 h (0.5 + 24 h), Zn was mostly presented in the vascular tissues (Figure 3).

Overall, these results demonstrate that the changes in the concentration and distribution of foliar absorbed Zn occurs quickly, with the Zn initially located in the adaxial epidermal layers (0–3 h) but later also moving to the middle (inner) vascular tissues of the leaf (24 h) which appear to correspond to the horizontal BSEs (Figure 3A). These observations for the cross-sections are in agreement with the data obtained from the repeated scanning of living leaves (Figure 2) which showed that the Zn translocation rate was limited in the first 6 h, with this stage likely corresponding to Zn accumulating in the epidermal layers, whereas after 24 h, the Zn had begun moving to the BSEs and thus the translocation rate increased thereafter.

### Identification of Differentially Expressed Genes in Sunflower Leaves at Different Zn Translocation Stages

Based on the XFM analyses, the following leaf samples were selected for transcriptome analysis: (i) L0, leaves without any treatment as the control; (ii) L1, leaves to which Zn had foliar applied for 0.5 h, representing the stage when Zn is mainly located in the epidermal layers; and (iii) leaves to which had kept growing for 24 h after 0.5 h of foliar Zn application, representing the stage when Zn is mainly located in the middle layer of the leaf. High-throughput Illumina sequencing yielded an average of 45.5 million clean reads from L0, 46.3 million from L1, and 46.3 million from L2 (Supplementary Table 2). Aligning to the genome of sunflower, L0 had a unique alignment rate of 86.1%, L1 of 85.7%, and L2 of 85.4% (Supplementary Table 3). The Spearman correlation coefficient (SCC) between the biological replicates of the three samples varied from 0.90 to 0.98 (Supplementary Table 4). The RT-qPCR analysis of the 15 randomly selected genes all showed a similar gene expression pattern with the transcriptome analysis (Supplementary Figure 4). Together, these findings indicate the good quality of the sequential data and replicates.

**FIGURE 4:**
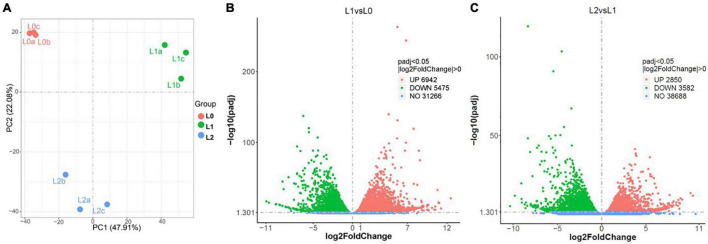
Overview of the sunflower leaf transcriptome analysis results. **(A)** Principal component analysis using the fragments per kilobase of transcript per million (FPKM) value of L0 (the leaves without Zn treatment), L1 (the leaves to which Zn applied for 0.5 h before being removed and then harvested immediately), and L2 (the leaves harvested 24 h after Zn applied for 0.5 h and removed), with a, b, and c referring to three replicates. **(B,C)** DEGs between L1 vs. L0 **(B)** and L2 vs. L1 **(C)**.

To compare gene expression among the three samples (L0, L1, and L2), the genes of the unique map libraries were all normalized to the FPKM value (Supplementary Additional File 1). Principal component analysis using the FPKM value showed that the leaf gene expression was significantly different among the three samples (Figure 4A). Specifically, L1 had 12,417 DEGs, with 6,924 genes upregulated and 5,475 downregulated compared to L0. Compared to L1, L2 had 2,850 genes upregulated and 3,582 genes downregulated (Figures 4B,C and Supplementary Additional File 2).

### Gene Ontology and Kyoto Encyclopedia of Genes and Genomes Enrichment Analysis of the Differentially Expressed Genes

The GO enrichment analysis was conducted for the DEGs of L1 vs. L0 and L2 vs. L1, with the most significantly enriched GO terms shown in Table 1 (full list in Supplementary Additional File 3). Comparing L1 to L0, the GO terms of “hydrolase activity: acting on glycosyl bonds,” “transferase activity: transferring acyl groups other than amino-acyl groups,” “cell periphery,” and “cell wall” were found to be upregulated, with these four GO terms all relevant to cell wall construction (Lopez-Casado et al., 2008). The GO terms of “carbohydrate binding” and “iron ion binding” were also upregulated, with these potentially relevant for the binding of the absorbed Zn^2+^. In addition, “response to wounding” was also significantly upregulated, suggesting that abiotic stress was induced by the foliar ZnSO_4_ application. In contrast, genes involved in “structural constituent of ribosome” and “protein transport” were downregulated. Comparing L2 to L1, the GO terms which were downregulated in L1 compared to L0, such as “structural constituent of ribosome” and “translation” were actually upregulated in L2. The reverse was also found, that some terms that were upregulated in L1 (compared to L0) such as “transferase activity: transferring acyl groups other than amino-acyl groups” and “response to wounding” were actually downregulated in L2 compared to L1. These observations suggest that the stresses that were induced immediately following the foliar application of ZnSO_4_ for 0.5 h had then subsided after 24 h.

**TABLE 1:** Selected significantly enriched GO terms of the DEGs in L0 (control leaves without Zn), L1 (leaves to which Zn was applied for 0.5 h before being removed and then harvested immediately), and L2 (leaves to which Zn was applied for 0.5 h before being removed and then left for 24 h before being harvested).

Category	GO ID	Description	*p*adj	Count

Up-regulated in L1 vs. L0				
MF	GO:0016798	Hydrolase activity: acting on glycosyl bonds	2.67E-07	120
MF	GO:0016747	Transferase activity: transferring acyl groups other than amino-acyl groups	1.39E-05	77
MF	GO:0030246	Carbohydrate binding	0.000204	59
CC	GO:0071944	Cell periphery	0.000738	51
MF	GO:0004857	Enzyme inhibitor activity	0.000946	42
CC	GO:0005618	Cell wall	0.003643	30
BP	GO:0009611	Response to wounding	0.003779	9
MF	GO:0005506	Iron ion binding	0.019931	102

**Down-regulated in L1 vs. L0**				

BP	GO:0006412	Translation	1.20E-67	240
MF	GO:0003735	Structural constituent of ribosome	1.02E-72	201
BP	GO:0006082	Organic acid metabolic process	0.00024	99
BP	GO:0051188	Cofactor biosynthetic process	0.001849	39
BP	GO:0015031	Protein transport	0.01952	45
MF	GO:0003723	RNA binding	5.53E-17	131

**Up-regulated in L2 vs. L1**				

MF	GO:0003735	Structural constituent of ribosome	7.07E-27	101
BP	GO:0006412	Translation	1.99E-18	114
CC	GO:0048046	Apoplast	0.015166	12
MF	GO:0016762	Xyloglucan: xyloglucosyl transferase activity	0.017901	12
MF	GO:0043565	Sequence-specific DNA binding	0.001219	52

**down-regulated in L2 vs. L1**				

CC	GO:0098796	Membrane protein complex	0.00011	40
MF	GO:0030414	Peptidase inhibitor activity	0.001209	12
MF	GO:0016747	Transferase activity: transferring acyl groups other than amino-acyl groups	0.002933	42
BP	GO:0009611	Response to wounding	0.0052	7
BP	GO:0016567	Protein ubiquitination	0.0052	22
MF	GO:0022803	Passive transmembrane transporter activity	0.02162	17

*Terms of padj < 0.05 as significantly enriched; Category refers to the functional classification of the Gene Ontology (GO) terms: biological process (BP), cellular component (CC), and molecular function (MF); Count means the number of the DEGs assigned to this GO term.*

The significantly enriched KEGG pathways from the DEGs of L1 vs. L0 and L2 vs. L1 are shown in Supplementary Additional File 4. Firstly, the most significantly upregulated pathways in L1 compared to L0 are “glycerolipid metabolism,” “alpha-Linolenic acid metabolism,” “fatty acid degradation,” “cutin, suberine and wax biosynthesis,” and “plant-pathogen interaction.” The former four pathways belong to lipid metabolism which is likely relevant to the cuticular lipids biosynthesis of the cell wall (Reina-Pinto and Yephremov, 2009). In contrast, the most significantly downregulated pathways in L1 compared to L0 are ribosome and ribosome biogenesis in eukaryotes. Secondly, compared with L1, the pathways of the ribosome and ribosome biogenesis in eukaryotes were upregulated in L2, whereas pathways such as alpha-Linolenic acid metabolism, glycerolipid metabolism, and plant hormone signal transduction were downregulated in L2.

### Differentially Expressed Genes Related to Zn Transporters and Metal Chelating Ligands

**FIGURE 5:**
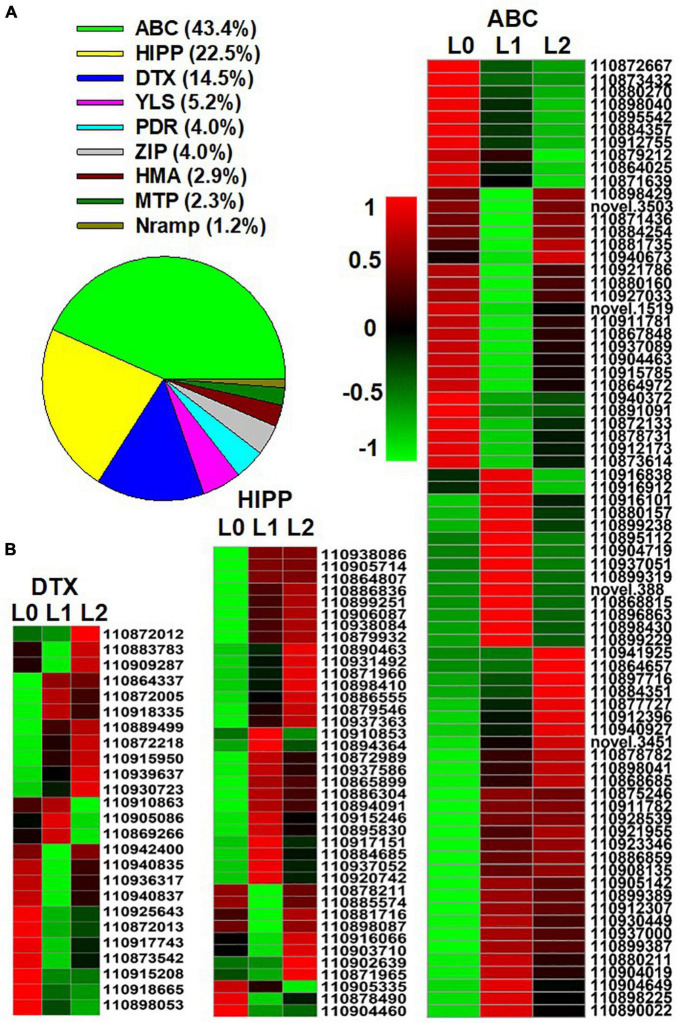
Genes of Zn transporters identified from the differentially expressed genes (DEGs) of the sunflower leaves. L0 = control leaves without Zn, L1 = leaves to which Zn was applied for 0.5 h before being removed and then harvested immediately, and L2 = leaves to which Zn was applied for 0.5 h before being removed and then left for 24 h before being harvested. **(A)** The proportion of the nine groups’ Zn transporters in the DEGs of the three samples. **(B)** Gene expression heatmap of the DEGs in the three Zn transporter families. ABC, ATP binding cassette transporters; HIPP, heavy metal-associated isoprenylated protein; DTX, protein DETOXIFICATION. Scale bar in B indicates a normalized FPKM value of genes.

After entering the leaf, the transmembrane transport of Zn is regulated by Zn transporters. A total of 173 genes belonging to the following nine groups of Zn transporters were found in the DEGs: ATP binding cassette (ABC) transporters, heavy metal-associated isoprenylated protein (HIPP), protein DETOXIFICATION (DTX), yellow stripe-like transporter (YSL), the ZRT/IRT-like protein (ZIP), pleiotropic drug resistance protein (PDR), metal tolerance proteins (MTP), heavy metal ATPase (HMA), and natural resistance-associated macrophage protein (NRAMP) (Figure 5A; the full list appears in Supplementary Additional File 5). Of the nine groups, ABC (43.3%), HIPP (22.5%), and DTX (14.5%) accounted for 80.3%, as shown by the expression heatmap (Figure 5B).

**FIGURE 6:**
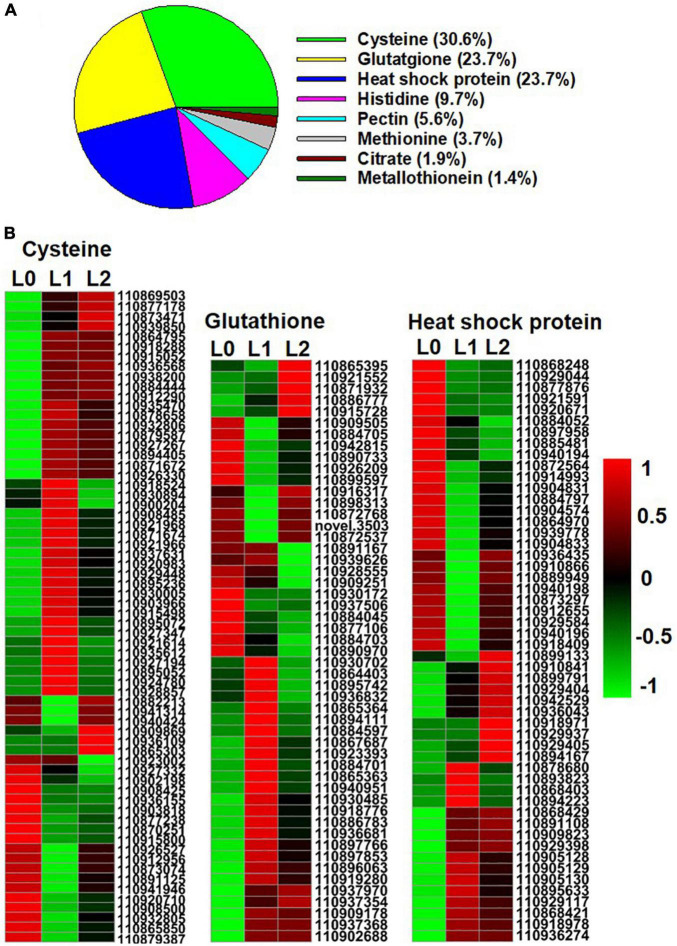
Genes of Zn metal chelating ligands identified from the DEGs of the sunflower leaves. L0 = control leaves without Zn, L1 = leaves to which Zn was applied for 0.5 h before being removed and then harvested immediately, and L2 = leaves to which Zn was applied for 0.5 h before being removed and then left for 24 h before being harvested. **(A)** The proportion of genes of the eight group metal chelating ligands in the DEGs of the three samples. **(B)** Gene expression heatmap of the DEGs in cysteine, glutathione, and heat shock protein. Scale bar in B indicates a normalized FPKM value of genes.

Previous studies have shown that when ZnSO_4_ is applied, the Zn is absorbed as Zn^2+^ (Li et al., 2018b). In contrast, Zn is present mainly in chelated forms within plant tissues given that high concentrations of Zn^2+^ are harmful to the plant’s normal metabolism (Sinclair and Krämer, 2012). In this regard, the present study identified eight groups of metal chelating ligand genes in the DEGs among the three samples, including genes related to cysteine, glutathione, heat shock protein (HSP), histidine, pectin, methionine, citrate, and metallothionein (MT) (Figure 6A and Supplementary Additional File 6). There was a total of 216 DEGs, of which genes related to cysteine accounted for 30.6%, while genes related to glutathione and heat shock protein (HSP) both accounted for 23.7%, suggesting that these three groups of chelating ligand played important roles in chelating the foliar absorbed Zn^2+^ in the leaf. The gene expression heatmap of the three groups is shown in Figure 6B.

## Discussion

### Distribution of Zn in Sunflower Leaves Following Foliar Zn Application

To explore the translocation of Zn following its foliar absorption in sunflower, the present study initially examined changes in the concentration and distribution of Zn within sunflower leaves following its foliar application. Firstly, bulk analyses of leaf Zn concentrations showed that foliar application of Zn for 0.5 h before being removed increased the leaf Zn concentration from ca. 90 mg kg^–1^ to ca. 600 mg kg^–1^, with no marked changes in these concentrations in subsequent 7 days (Figure 1). Next, to examine changes in the distribution of the Zn within the sunflower leaf, synchrotron-based XFM was used for *in situ* analyses. It was found that the Zn within the interveinal leaf areas was gradually translocated into the veins, especially during the first 6 h (Figure 2). Furthermore, XFM analysis of the Zn distribution in leaf cross-sections showed that the Zn in the upper epidermal layer was quickly moved to the lower epidermis, with these epidermal layers being the main site for Zn accumulation within the first 3 h following foliar Zn application. Combining these observations with the findings in Li et al. (2019), who showed that foliar-absorbed Zn was located in the apoplast of the epidermal cells and that the BSEs are important in Zn translocation in sunflower leaves, it is likely that this Zn that is accumulating in the epidermal cells is also likely to be in the apoplast, and that the movement of the Zn between the epidermal layers is likely *via* the BSEs. When examined at a period of 24 h after the foliar application of the Zn, much of the Zn had moved from the epidermal layers to the inner vascular tissues, especially the horizontal BSEs (Figure 3). Our observation that the majority of the Zn remained within the leaf over a period of 7 days (as opposed to being translocated to other tissues) is in accordance with previous studies which found that the foliar absorbed Zn has limited mobility in plants (Zhang and Brown, 1999; Du et al., 2015). Regardless, we show here that although there is only limited translocation out of the leaf, the distribution of the Zn within the leaf changes markedly following its absorption—the absorbed Zn initially moved from the upper epidermis to the lower epidermis, before gradually moving to the horizontal BSEs and veins (Figures 2, 3).

### Leaf Gene Expression During Zn Translocation Process and Implications for Foliar ZnSO_4_ Application

Not only did we examine changes in Zn distribution in leaves following its foliar application, but we also examined corresponding changes in leaf gene expression. It was found that genes related to stress response were upregulated rapidly following the 0.5 h foliar ZnSO_4_ application, including GO terms of response to wounding and protein ubiquitination (Dametto et al., 2015) and the KEGG pathway of plant-pathogen interaction. Furthermore, the mitogen-activated protein kinase (MAPK) signaling pathway was upregulated, and stress-related responses including leaf cell wall reinforcement and lipid metabolism were also significantly upregulated (Supplementary Additional Files 3, 4). In contrast, GO terms of translation, structural constituent of ribosome, and protein transport, were downregulated in L1 compared to L0, suggesting that the essential metabolic activity of the leaf had been impaired. These various observations suggest that the foliar application of Zn caused a stress reaction to the leaf. We have previously found that when ZnSO_4_ is applied to the leaf surface, the Zn enters the leaf as Zn^2+^ (Li et al., 2018a), but it is known that high concentrations of free Zn^2+^ within the leaf can cause toxicity due to the high affinity of Zn^2+^ for numerous proteins, with this resulting in metabolic disorders (Hessels and Merkx, 2015). Hence, it is likely that the stress observed in the present study following the foliar application of Zn likely resulted from the sudden fourfold increase in leaf Zn concentration following foliar application (Figure 1). Indeed, similar results were reported by Li et al. (2021) who found that the genes relevant to “response to wounding” in the NGTs of sunflower leaf were also significantly upregulated after foliar Zn application, suggesting that the foliar Zn application caused abiotic stress to the plant. This finding is also in agreement with previous studies that have reported that lower Zn application rates can result in higher efficiencies and that higher application rates can cause localized toxicity (Doolette et al., 2020).

Although the foliar application of Zn caused a toxic response, this stress was only temporary—the stress-relevant GO terms which were upregulated rapidly after Zn application (L1) were subsequently downregulated after 24 h (L2), while those that were downregulated in L1 were upregulated in L2 (Supplementary Additional File 3). Accordingly, Zn was found mainly in the epidermal layers (including the NGTs) in the first 3 h (Figure 3), with this likely being a detoxification strategy (Frey et al., 2000; Tappero et al., 2007; Horeth et al., 2020; Li et al., 2021). In contrast, after 24 h, much of the Zn had moved to the horizontal BSEs and the veins. This stress alleviation was also related to Zn transporters and chelating ligands. For example, the ABC transporters (43.4%), HIPP (22.5%), and DTX (14.5%) have been previously reported to be related to the alleviation of Zn and Cd stress (Moons, 2003; Dong et al., 2019; Manara et al., 2020), with ABC transporters playing a role in the compartmentalization of excess metals in vacuoles (Sharma et al., 2016). Furthermore, of the eight groups of chelating ligands, heat shock protein (23.7%) is also related to metal stress alleviation (Hasan et al., 2017). However, it must be noted that in the present study, Zn was applied to the leaves for only 0.5 h, and it is likely that a longer application time would result in increased absorption of Zn, and hence the resulting stress would presumably be prolonged. Therefore, care must be taken when devising a foliar fertilization regime to avoid causing severe leaf stress.

Given that the Zn was applied as ZnSO_4_, it is likely that the SO_4_^2–^ also entered the leaves. In this regard, it is known that S that is foliar-applied can be metabolized into cystine, methionine, and glutathione (Legris-Delaporte et al., 1987; Landry et al., 1991), and it is known that foliar application of S can be helpful for plant tolerance against heat stress (Waraich et al., 2022) and that root uptake of S can mitigate Cd toxicity (Adhikari et al., 2018). Hence, in the present study, changes in the leaf gene expression are due to both the Zn^2+^ and the SO_4_^2–^, although the effects of SO_4_^2–^ are unclear (i.e., whether it contributed to the stress or whether it assisted in alleviating the stress caused by Zn).

### Reasons for Limited Translocation of the Foliar Absorbed Zn and New Insights for Improving the Efficiency of Foliar Zn Fertilizer

The present study found that little of the foliar absorbed Zn moved out of the leaf to which it has been applied, even within 7 days (Figure 1), *in situ* analyses showed that the Zn appears to be mobile within the leaf, with the Zn found within the vascular tissues after 24 h (Figures 1–3). It has previously been hypothesized that the limited translocation of Zn out of the leaf is due to the binding of Zn to the epidermal cell wall, with this restricting further redistribution (Du et al., 2015). However, our results demonstrate that the main reason for the limited mobility of Zn is related to the translocation of Zn in vascular tissues. In this regard, the xylem is unlikely to be involved in Zn translocation from the leaf given that the xylem is for the upward movement of water and nutrients from roots to shoots, while the phloem is responsible for both the upwards and downwards movement of solutes. Indeed, it was found in wheat (*Triticum aestivum*) (Haslett et al., 2001) and citrus (*Citrus reticulatus*) (Du et al., 2020) that the translocation of the foliar absorbed nutrients occurred *via* the phloem. Tian et al. (2015) also reported for sunflower that foliar absorbed Zn was translocated within the phloem. Thus, it seems likely that the loading of Zn into the phloem, or the low mobility of Zn within the phloem itself, are responsible for the limited translocation of the foliar absorbed Zn in plants.

In this regard, the loading of Zn into the phloem requires Zn transporters, with the mobility of Zn within the phloem being influenced by Zn speciation (Ricachenevsky et al., 2015; Nozoye, 2018). The Zn transporters in sunflowers have not received much attention, with Huang et al. (2021) reporting that some HMA and MTP genes are involved in the Zn translocation in seeds of sunflowers. Of the DEGs related to Zn transporters in sunflower leaves, *HMA3* (gene ID: 110878461) was significantly upregulated in L1 and L2 compared to L0, and hence it could potentially play a role in the translocation of Zn into the vacuoles for detoxification, as discussed for other plant species (Cai et al., 2019); In contrast, *NRAMP3* (gene ID: 110889223) was downregulated in L1 compared to L0 which could be involved in the Zn translocation out of the vacuoles (Thomine et al., 2003); and *YSL1* (gene ID: 110924134) was downregulated in L1 while upregulated in L2 which could be related to Zn loading into the vascular tissues (Sinclair and Krämer, 2012; Supplementary Additional File 5). In addition, of the eight groups of metal-chelating ligands identified from the DEGs among L0, L1, and L2, cysteine and glutathione were the two main groups (Figure 6). This observation suggests that cysteine and glutathione may be important in chelating Zn^2+^ in sunflower leaves and that their increase in the leaf might enhance the translocation of the foliar absorbed Zn (Palmer and Stangoulis, 2018; Nakamura et al., 2019; Wongkaew et al., 2019). In order to improve the efficiency of foliar Zn fertilizers, future studies should investigate which Zn transporters are involved in the Zn translocation in leaves and what Zn speciation favors Zn translocation in the leaves.

### A New Methodological System for Studying Element Translocation in Plant

Our combined use of XFM together with transcriptome analysis illustrates the ability of this methodological platform to allow the systemic study of nutrient translocation in plants. Given that changes in gene expression can be rapid and XFM can provide *in situ* elemental imaging at high temporal and spatial resolutions, this system can provide information on the physiological behavior coupled with molecular activity. Such information is important in bridging the phenotype to the genotype gap.

### Translocation of the Foliar Absorbed Zn in Sunflower Leaves

**FIGURE 7:**
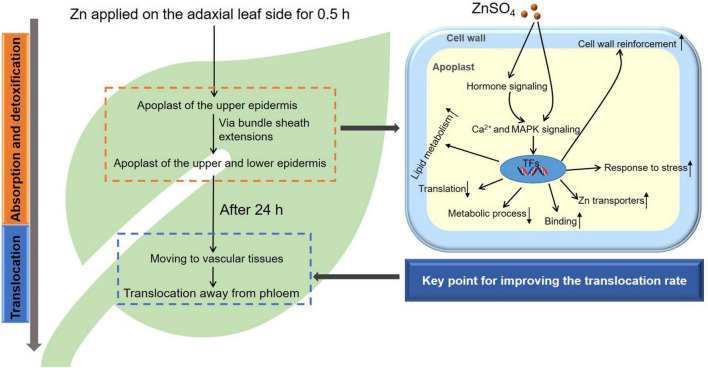
The proposed translocation process of the foliar absorbed Zn in sunflower leaves, with the metabolic activity in the leaf deciphered based on the transcriptome analysis.

In conclusion, we found that foliar application of Zn caused rapid stress to the sunflower leaf, with the foliar Zn absorption process accompanied by the detoxification process (Figure 7). Correspondingly, Zn was initially presented in the apoplast of the upper and lower epidermal layers (including in the NGTs) as a detoxification strategy, and genes within the leaves involved in cell wall reinforcement, response to stress, and Zn transporters were upregulated to address this stress (Figures 2, 3, 7 and Table 1). After this stress receded, Zn was gradually moved to the vascular tissues, and the further translocation was likely *via* the phloem. It seems that the limited translocation of Zn in the phloem (for example, limited loading into the phloem, or limited mobility in the phloem) was the key reason for the restricted translocation of the foliar absorbed Zn in leaves of sunflower.

## Data Availability Statement

All data supporting the findings of this study are available within the paper, the supplementary information and additional file published online. The RNA sequencing data can be found in NCBI database (accession: PRJNA723486).

## Author Contributions

CL: conceptualization, data curation, formal analysis, investigation, methodology, writing—original draft, review and editing, and funding acquisition. LiW: formal analysis, investigation, and methodology. JW and FB: investigation, methodology, and writing—review and editing. NW, YC, YY, and LeW: methodology. DP: resources and methodology. TR, PW, and EL: writing—review and editing. YW: funding acquisition, supervision, and writing—review and editing. PK: conceptualization, data curation, formal analysis, investigation, methodology, writing original draft and review and editing, funding acquisition, and supervision. All authors contributed to the article and approved the submitted version.

## Conflict of Interest

The authors declare that the research was conducted in the absence of any commercial or financial relationships that could be construed as a potential conflict of interest.

## Publisher’s Note

All claims expressed in this article are solely those of the authors and do not necessarily represent those of their affiliated organizations, or those of the publisher, the editors and the reviewers. Any product that may be evaluated in this article, or claim that may be made by its manufacturer, is not guaranteed or endorsed by the publisher.

## Supplementary Material

Supplementary Additional File 5 List of the zinc (Zn) transporter genes identified from the DEGs.

Supplementary Additional File 6 List of the metal-chelating ligand genes identified from the DEGs.
