# Supplementary material for: Translocation of Foliar Absorbed Zn in Sunflower (Helianthus annuus) Leaves
Source: Front Plant Sci. 2022 Mar 2;13:757048. doi: 10.3389/fpls.2022.757048 (PMC8924476; doi:10.3389/fpls.2022.757048)
Supplement: Supplementary file 1 [file Data_Sheet_1.docx]

Supplementary Material

**Translocation of foliar absorbed Zn in sunflower (*Helianthus annuus*) leaves**

Cui Li^1*^, Linlin Wang^1^, Jingtao Wu^2^, F. Pax C. Blamey^3^, Nina Wang^1^, Yanlong Chen^1^, Yin Ye ^1^, Lei Wang^1^, David J. Paterson^4^, Thea L. Read^5^, Peng Wang^6^, Enzo Lombi^5^, Yuheng Wang^1*^, Peter M. Kopittke^3^

^1^Northwestern Polytechnical University, School of Ecology and Environment, Xi’an 710072, China. ^2^Chinese Academy of Sciences, South China Botanical Garden, Key Laboratory of Vegetation Restoration and Management of Degraded Ecosystems, Guangzhou 510650, China. ^3^The University of Queensland, School of Agriculture and Food Sciences, St Lucia, Queensland, 4072, Australia. ^4^Australian Synchrotron, Clayton, Victoria, 3168, Australia. ^5^University of South Australia, Future Industries Institute, Mawson Lakes, South Australia, 5095, Australia. ^6^Nanjing Agricultural University, College of Resources and Environmental Sciences, Nanjing, 210095, China.


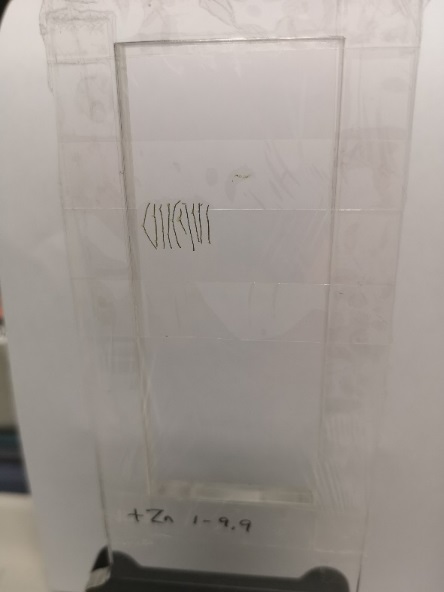


Figure S1 A photo showing how the freeze dried sunflower leaf cross sections were mounted on the sample holder for XFM analysis.


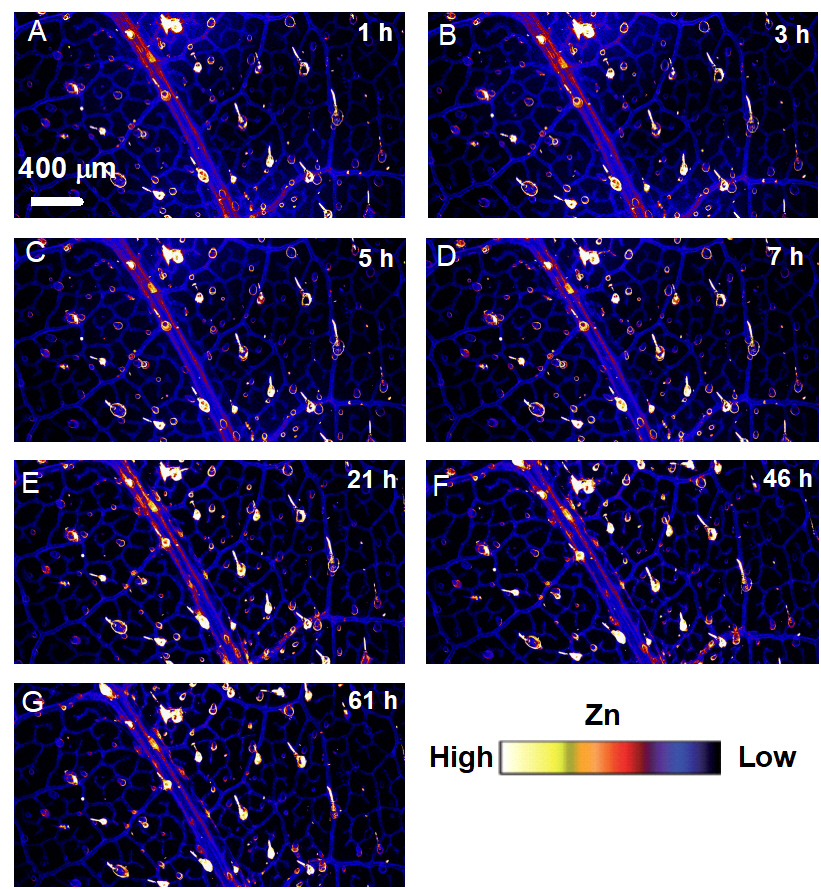


Figure S2 XFM images of the Replicate 2 of Figure 2. The XFM scans were done at 1 h (A), 3 h (B), 5 h (C), 7 h (D), 21 h (E), 46 h (F), and 61 h (G) respectively after 0.5 h foliar Zn application. Colors are comparable in (A-G). The scale in (A) applies to (B-G).


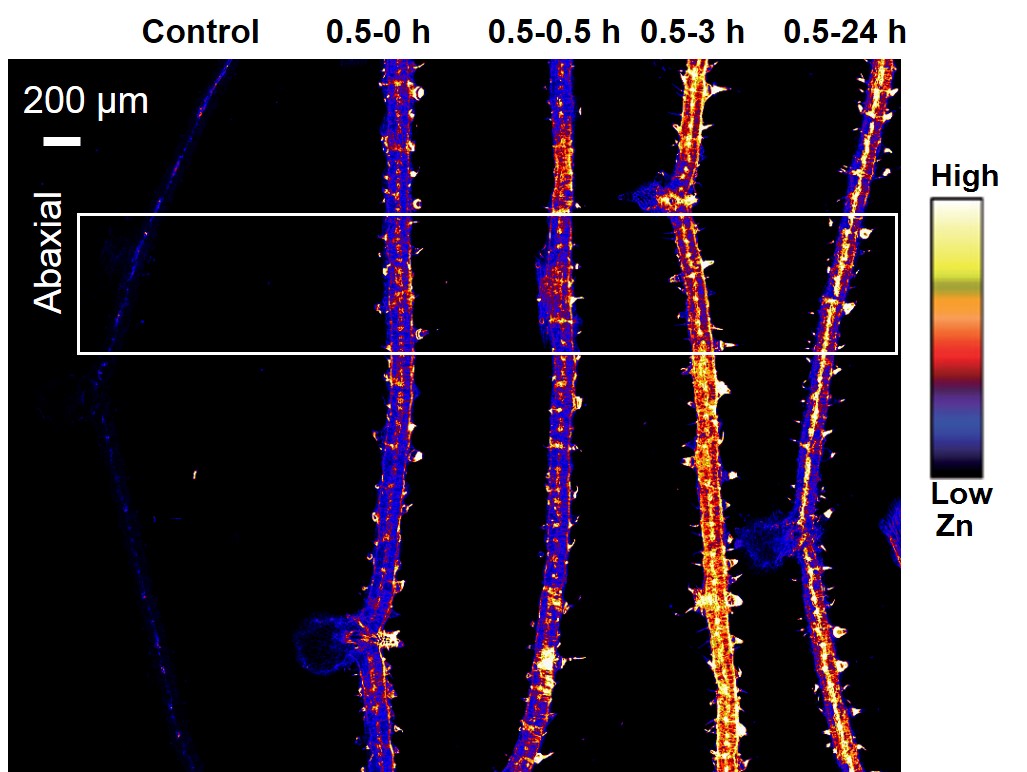


Figure S3 Replicate 2 of Figure 3. XFM image showing Zn distribution in sunflower leaf cross sections of control and at 0, 0.5, 3 and 24 h following 0.5 h foliar Zn application. Data of Replicate 2 in Figure 3c is from the white box region.


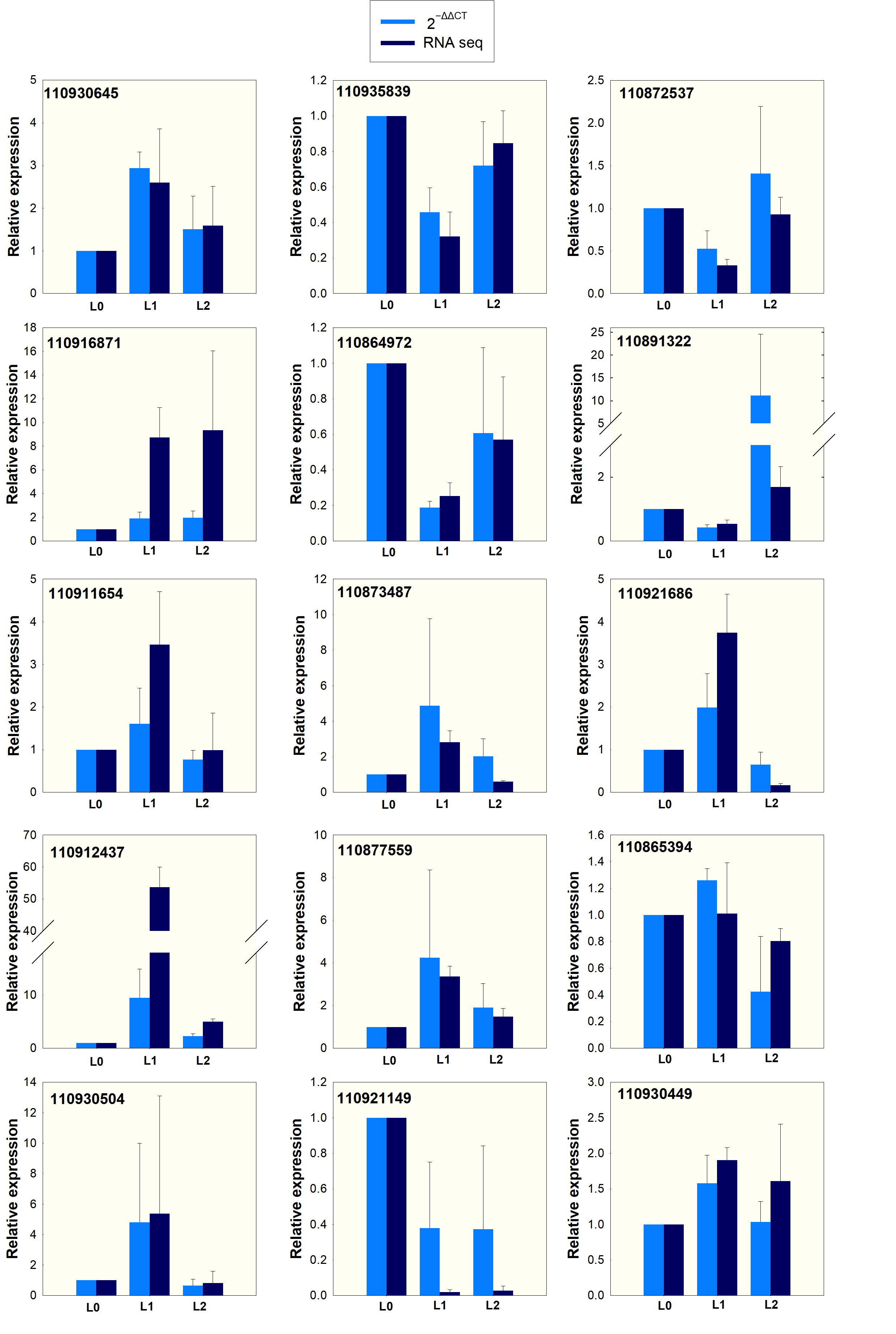


Figure S4 Comparison of gene expression pattern of the RT-qPCR results (2^−ΔΔCT^) and the transcriptome results (RNA seq) of the random selected 15 genes. Expression is relative to L0, with the value of which set as 1, data are means ± SD.

Table S1 Primers used for RT-qPCR

| Gene ID | Gene Description | Accession number | Forward | Reverse |
| --- | --- | --- | --- | --- |
| 110930645 | uncharacterized | XM_022173993.2 | AATGAGCAGAAGGCGTGGAA | GGGCTGTGCATAGTCCATGA |
| 110916871 | uncharacterized | XM_022161525.2 | CCTTCATCGGTGTAGGACGG | CACAGCCGACATTTTCCCAT |
| 110911654 | putative receptor protein kinase ZmPK1 | XM_022156274.2 | AGCGAGGTTGTGAAGATGCT | ATCGAGTCACAGCCAAGTGT |
| 110912437 | protein LHY | XM_035984220.1 | ATCAACGGCTACTGTTGGCA | AGAAGTAATCCTCACCAAAATGAGC |
| 110930504 | short-chain dehydrogenase TIC 32, chloroplastic | XM_022173817.2 | ATCGGTTCGTTGATTCTGGGT | ACCTTTCTTGACAGCGGAAGA |
| 110935839 | CBL-interacting serine/threonine-protein kinase 5 | XM_022178191.2 | AGTATGAGTTTCCGCCGTGG | TCTCATGATCGCCGGAATGG |
| 110864972 | ABC transporter I family member 19-like | XM_022114143.2 | TGTTGTGCGGGTGCTAGATT | GGTCGATACCTTCAACGCCA |
| 110873487 | probable sodium/metabolite  cotransporter BASS3 chloroplastic | XM_022122429.2 | CCCCACCTTTCAGTAACCCC | CCACCCCACTTTTCCGATCA |
| 110877559 | organic cation/carnitine transporter 7-like | XM_022125713.2 | ATCGGTGCTGGTTTAGGGTG | GGATAGTCCGAGCAGCCATC |
| 110921149 | adagio protein 3-like transcript variant X3 | XM_022165425.1 | TCTCGGTTTACGGGAGGACT | GCATTCCAAAACCCGCCATT |
| 110872537 | glutathione S-transferase DHAR2-like | XM_022121348.2 | TACTCCCCCTGAACTTGCCT | GGCTGTGACCTTTTCTCCGT |
| 110891322 | metallothionein-like protein 1 | XM_022139011.2 | CTGCAACGGAAAGTGTGGCT | ATCTTCTTTGGCGCAACGC |
| 110921686 | beta-amylase 3, chloroplastic | XM_022166039.2 | GGGGAAAATCCGGTCCACAT | TTGCCGGAGTACCATTGCAT |
| 110865394 | probable glutathione S-transferase | XM_022114641.2 | TACAGGGAGTCCGTTCGTCT | CGGATTCCCGTTGTGTACGA |
| 110930449 | ABC transporter C family member 10-like | XM_035987671.1 | GCGTTTACGGCTTTGTTGGT | AGCTGCCTTCTGGAAGTGAC |
| 110936586 | ubiquitin | XM_022178991.2 | AGCAAAGGCTCATCTTTGCAGG | GATAGTGTCCGAGCTCTCCA |
| 110903735 | actin | XM_022149519.2 | AGGGCGGTCTTTCCAAGTAT | ACATACATGGCGGGAACATT |

Table S2 Overview of RNA sequential results and data quality assessment

| Sample | Raw reads | Clean reads | Clean  Bases (GB) | Error  Rate (%) | Q20 (%) | Q30 (%) | GC content (%) |
| --- | --- | --- | --- | --- | --- | --- | --- |
| L0a | 46152194 | 45434662 | 6.82 | 0.03 | 97.91 | 93.98 | 44.91 |
| L0b | 46005636 | 45527934 | 6.83 | 0.03 | 97.84 | 93.84 | 44.54 |
| L0c | 46315754 | 45532944 | 6.83 | 0.03 | 98.00 | 94.16 | 44.95 |
| L0 mean | 46157861 | 45498513 | 6.83 | 0.03 | 97.92 | 93.99 | 44.80 |
|  |  |  |  |  |  |  |  |
| L1a | 46883476 | 46339246 | 6.95 | 0.03 | 97.97 | 94.06 | 44.73 |
| L1b | 46110624 | 45674032 | 6.85 | 0.03 | 97.83 | 93.65 | 44.75 |
| L1c | 47267584 | 46760708 | 7.01 | 0.03 | 97.88 | 93.86 | 45.12 |
| L1 mean | 46753895 | 46257995 | 6.94 | 0.03 | 97.89 | 93.86 | 44.87 |
|  |  |  |  |  |  |  |  |
| L2a | 45659378 | 45179664 | 6.78 | 0.03 | 97.74 | 93.55 | 44.40 |
| L2b | 47633620 | 47156054 | 7.07 | 0.03 | 97.83 | 93.78 | 44.59 |
| L2c | 47041112 | 46574898 | 6.99 | 0.03 | 97.81 | 93.76 | 44.42 |
| L0 mean | 46778037 | 46303539 | 6.95 | 0.03 | 97.79 | 93.70 | 44.47 |

Q20 and Q30 refer to bases whose *P*hred>20 or 30 accounting to the total bases.

Table S3 Mapping rates of the clean reads aligned to the genome of sunflower

| Sample | Unique map  (%) | Multi map  (%) | Read1 map  (%) | Read2 map (%) | Positive map (%) | Negative map (%) |
| --- | --- | --- | --- | --- | --- | --- |
| L0a | 86.41 | 3.94 | 43.34 | 43.08 | 43.14 | 43.27 |
| L0b | 85.40 | 4.07 | 42.86 | 42.54 | 42.64 | 42.76 |
| L0c | 86.51 | 4.00 | 43.38 | 43.12 | 43.19 | 43.32 |
| L0 mean | 86.11 | 4.00 | 43.19 | 42.91 | 42.99 | 43.12 |
| L1a | 85.89 | 3.95 | 43.07 | 42.82 | 42.88 | 43.01 |
| L1b | 85.92 | 3.98 | 43.09 | 42.83 | 42.89 | 43.03 |
| L1c | 85.23 | 5.03 | 42.76 | 42.47 | 42.55 | 42.68 |
| L1 mean | 85.68 | 4.32 | 42.97 | 42.71 | 42.77 | 42.91 |
| L2a | 85.71 | 3.82 | 43.03 | 42.68 | 42.79 | 42.91 |
| L2b | 84.96 | 3.74 | 42.65 | 42.31 | 42.42 | 42.53 |
| L2c | 85.57 | 3.91 | 42.93 | 42.64 | 42.73 | 42.84 |
| L2 mean | 85.41% | 3.82% | 42.87% | 42.54% | 42.65% | 42.76% |

Table S4 Pearson correlation (R^2^) between samples (calculated based on the FPKM values of each sample)

|  | L0a | L0b | L0c | L1a | L1b | L1c | L2a | L2b | L2c |
| --- | --- | --- | --- | --- | --- | --- | --- | --- | --- |
| L0a | 1 | 0.967 | 0.982 | 0.84 | 0.809 | 0.788 | 0.876 | 0.898 | 0.869 |
| L0b | 0.967 | 1 | 0.967 | 0.832 | 0.814 | 0.794 | 0.88 | 0.886 | 0.866 |
| L0c | 0.982 | 0.967 | 1 | 0.84 | 0.809 | 0.789 | 0.876 | 0.898 | 0.868 |
| L1a | 0.84 | 0.832 | 0.84 | 1 | 0.942 | 0.924 | 0.846 | 0.851 | 0.857 |
| L1b | 0.809 | 0.814 | 0.809 | 0.942 | 1 | 0.937 | 0.856 | 0.818 | 0.874 |
| L1c | 0.788 | 0.794 | 0.789 | 0.924 | 0.937 | 1 | 0.825 | 0.773 | 0.851 |
| L2a | 0.876 | 0.88 | 0.876 | 0.846 | 0.856 | 0.825 | 1 | 0.909 | 0.954 |
| L2b | 0.898 | 0.886 | 0.898 | 0.851 | 0.818 | 0.773 | 0.909 | 1 | 0.895 |
| L2c | 0.869 | 0.866 | 0.868 | 0.857 | 0.874 | 0.851 | 0.954 | 0.895 | 1 |
